# The Protective Effect of FOXO3 rs2802292 G-Allele on Food Intake in a Southern Italian Cohort Affected by MASLD

**DOI:** 10.3390/nu17081315

**Published:** 2025-04-10

**Authors:** Rossella Donghia, Elisabetta Di Nicola, Rossella Tatoli, Giovanna Forte, Martina Lepore Signorile, Caterina Bonfiglio, Marialaura Latrofa, Katia De Marco, Andrea Manghisi, Vittoria Disciglio, Candida Fasano, Paola Sanese, Filomena Cariola, Antonia Lucia Buonadonna, Gianluigi Giannelli, Valentina Grossi, Cristiano Simone

**Affiliations:** 1 Data Science Unit, National Institute of Gastroenterology, IRCCS “Saverio de Bellis” Research Hospital, 70013 Castellana Grotte, Italy; rossella.donghia@irccsdebellis.it (R.D.); rossella.tatoli@irccsdebellis.it (R.T.); catia.bonfiglio@irccsdebellis.it (C.B.); 2 Medical Genetics, National Institute of Gastroenterology, IRCCS “Saverio de Bellis” Research Hospital, 70013 Castellana Grotte, Italy; elisabetta.dinicola@irccsdebellis.it (E.D.N.); giovanna.forte@irccsdebellis.it (G.F.); martina.lepore@irccsdebellis.it (M.L.S.); marialaura.latrofa@irccsdebellis.it (M.L.); katia.demarco@irccsdebellis.it (K.D.M.); andrea.manghisi@irccsdebellis.it (A.M.); vittoria.disciglio@irccsdebellis.it (V.D.); candida.fasano@irccsdebellis.it (C.F.); paola.sanese@irccsdebellis.it (P.S.); filo.cariola@irccsdebellis.it (F.C.); lucia.buonadonna@irccsdebellis.it (A.L.B.); 3 Scientific Direction, National Institute of Gastroenterology, IRCCS “Saverio de Bellis” Research Hospital, 70013 Castellana Grotte, Italy; gianluigi.giannelli@irccsdebellis.it; 4 Medical Genetics, Department of Precision and Regenerative Medicine and Jonic Area (DiMePRe-J), University of Bari Aldo Moro, 70124 Bari, Italy

**Keywords:** MASLD, food intake, single nucleotide polymorphism

## Abstract

**Background**: Metabolic Dysfunction-Associated Steatotic Liver Disease (MASLD) is a cluster of conditions characterized by accumulations of fat, metabolic factors such as obesity, diabetes and high cholesterol. MASLD is now the leading cause of chronic liver disease worldwide, with a rapidly increasing trend. We aimed to demonstrate that genetic variants of rs2802292 SNP can influence the development of MASLD even after many years. **Methods**: We studied 650 participants from the NUTRIHEP cohort, both at baseline (2005–2006) and at first recall (2014–2018), and genotyped rs2802292. The validated European Prospective Investigation into Cancer and Nutrition (EPIC) questionnaire was administered during the visit, and each single food was assigned to one of 33 groups. **Results**: Associations of food intake at baseline with MASLD were found in the first recall, for each genotype, GG, GT, and TT, and several covariates were used to adjust models. Dressing fats other than olive oil resulted protection against MASLD in GG subjects, whereas seed oil, juices, and spirits resulted in protection against MASLD for GT subjects. An increased risk of MASLD was found for subjects with the TT genotype for white meat intake (OR = 1.018, *p* = 0.031, 1.002 to 1.035 95% C.I.), ready-to-eat dishes (OR = 1.015, *p* = 0.033, 1.001 to 1.029 95% C.I.), processed meat (OR = 1.093, *p* = 0.003, 1.031 to 1.158 95% C.I.), and processed fish (OR = 1.085, *p* = 0.037, 1.005 to 1.172 95% C.I.). **Conclusions**: Subjects with the TT genotype had a higher risk of developing MASLD than subjects with other genotypes. A healthier lifestyle is important to counteract liver disease.

## 1. Introduction

Proteins of the Forkhead box O (FOXO) family are transcription factors involved in both physiological and pathological processes, including cellular homeostasis, metabolism, oxidative stress response, and lifespan regulation [1]. Specifically, Forkhead box O3 (FOXO3) is widely expressed in various human organs and tissues, and its biological effects are determined by phosphorylation, which occurs in the nucleus, inhibiting its transcriptional activity, or the cytoplasm, promoting the regulatory effects on biological processes [2]. A wide range of diseases are linked to the above processes; specifically, oxidative stress response alterations are involved in the development of cancer, diabetes, Alzheimer’s disease, and atherosclerosis, along with other respiratory and kidney diseases [3]. Oxidative stress contributes to disease through two major mechanisms: the production of reactive species, which directly oxidize macromolecules, leading to cell function alterations and death [4], and an aberrant redox signaling, where oxidants act as second messengers [5]. Oxidative stress occurs through an imbalance between oxidants and antioxidant species, which may damage biological systems [5]. FOXO3 was shown to play a crucial role in maintaining cellular homeostasis by promoting the transcription of genes, such as Superoxide Dismutase and Catalase, which enhance the antioxidant potential of cells and regulate oxidative stress [6]. Moreover, the nutrient and energy sensor AMP-activated protein kinase (AMPK) directly phosphorylates mammalian FOXO3, leading to the activation of its transcriptional activity and the regulation of gene expression programs that control the energy balance [7]. Other studies characterized the glucose and lipid metabolism by knocking out FOXO3 and FOXO1 from mouse liver and found that blood glucose levels were decreased, while serum triglyceride and cholesterol concentrations were increased. Specifically, FOXO3 knockdown increased the expression of genes related to triglycerides synthesis, including SREBP1c [8]. These data suggest that FOXO3 inactivation could be a potential mechanism by which insulin reduces hepatic glucose production and increases hepatic lipid synthesis and secretion, causing hepatosteatosis [9]. Moreover, it was found that a variety of dietary restriction regimens extend both lifespan and healthspan [10]. Studies revealed the involvement of the FOXO ortholog Daf-16 in the life-extending effect of dietary restriction [11]. Since stress resistance is highly correlated with lifespan extension, the relocalization of FOXO factors within the nucleus after stress stimuli allows an adaptive response [12]. Overall, these data suggest that FOXO3 could be involved in oxidative stress responses, metabolism regulation, and dietary outcomes. Other studies identified the effects of some compounds as comparable to dietary restriction, as they promote physiological functions, lifespan increase [13], and decreased pathological outcomes [14,15]. These compounds, such as metformin, rapamycin, and aspirin, are known as caloric restriction mimetics (CRMs) and promote protective pathways such as autophagy, which is involved in cytoplasmic recycling, counteracting age-related processes [16]. For instance, a recent preclinical study in mice revealed that 3-month rapamycin administration improved health status and increased lifespan [17]. In addition, several case-control studies have shown associations between FOXO3A gene polymorphisms and human longevity [18,19,20]. A meta-analysis of existing studies identified a correlation between the rs2802292 single nucleotide polymorphism (SNP) of FOXO3 and lifespan extension [21]. We recently found that the intronic rs2802292 G-allele was correlated with a higher expression of FOXO3, suggesting that it might be a regulatory region. Specifically, we showed that the rs2802292 G-allele created a novel HSE binding site for HSF1 in a 90 bp sequence, which induced FOXO3 expression, both at protein and mRNA levels, in response to oxidative stress, heat shock, and glucose restriction [22]. Notably, we also found protective effects of the G-allele of FOXO3 against diabetes and liver failure [23].

MASLD is a liver disorder defined by the accumulation of fat in more than 5% of hepatocytes, the presence of one or more cardiometabolic risk factor(s), and a wrong life style [24]. It covers a spectrum of hepatic diseases, ranging from simple steatosis to non-alcoholic steatohepatitis (NASH), which can evolve to cirrhosis, liver failure, and hepatocellular carcinoma [25]. Chronic inflammation and oxidative stress are two of the factors that contribute to the onset and progression of MASLD [26].

MASLD is closely associated with obesity, insulin resistance and type 2 diabetes, with which it shares many pathogenetic features [27]; therefore, it has been recognized as an important risk factor for several diseases, including cardiovascular and other cardiac complications, hepatocellular carcinoma (HCC), extrahepatic malignancies, and chronic kidney disease [28,29] negatively impacting the quality of life. Currently, MASLD affects approximately 25% of the world’s adult population, with different distributions across geographic areas, ethnicity, and socioeconomic and lifestyle factors [30,31], significantly impacting patient outcomes and health sectors [32]. Furthermore, it would be appropriate to promote adequate education globally that teaches a correct lifestyle in order to prevent this condition [33].

Several studies support the view that it is the close interaction between genetic and environmental factors, including diet, that promotes the development of inflammation, oxidative stress, lipid peroxidation, and mitochondrial dysfunction, promoting the development of MASLD [34]. Moreover, several studies revealed that hepatic steatosis, inflammation, and risk of fibrosis are influenced by genetic variants, diet and their interactions. These data suggest that FOXO3 is a potential target that could be used to protect subjects at increased risk.

Based on these findings, in this longitudinal study, we evaluated the effect of FOXO3 rs2802292 G-allele on dietary intake, considering the outcomes of genetic and diet interactions from the perspective of pathological risks.

## 2. Materials and Methods

### 2.1. Study Population

As described in a previous paper [23], the study cohort named NUTRIHEP was first established in 2005–2006 in the municipality of Putignano (subjects aged > 18 years) (Bari), a small city in southern Italy. Participants were first interviewed (*n* = 2550) in 2004–2005 by trained physicians to collect information about their sociodemographic, clinical characteristics and dietary habits [35], and then in the years 2014–2018, all eligible subjects continued follow-up with NUTRIHEP 2, which achieved 86.08% compliance (*n* = 2195). From these patients, 650 samples were stored for future molecular analysis; these are included in this study [23]. All participants signed informed consent after receiving complete information about their medical data to be studied. This study was approved by the Ethics Committee of the Ministry of Health (DDG-CE-502/2005; DDG-CE-792/2014, 20 May 2005 and 14 February 2014, respectively).

### 2.2. Lifestyle, Clinical, and Dietary Assessment

Lifestyle and anthropometric assessments were conducted by a physician during an interview at the study center. Education level was expressed as years of schooling. Weight was measured with an electronic scale (SECA©) and recorded to the nearest 0.1 kg. Height was measured with a wall-mounted stage meter (SECA©) and recorded to the nearest 1 cm. Body mass index (BMI) was calculated as kg/m^2^. Blood was collected from all subjects in the morning after overnight fasting, and aliquots were stored in the biobank according to validated protocols and processed by trained personnel. Diabetes was diagnosed based on drug treatment or consultation with an endocrinologist certifying the disease, while MASLD was built based on the diagnostic criteria of the Delphi consensus [36]. The validated Food Frequency Questionnaire from the European Prospective Investigation into Cancer and Nutrition (EPIC) was administered during the visit, and each food item (260 food items, organized in 15 sections) was converted into an average daily intake in grams.

The questionnaire is organized in major food groups: dry first courses; soups; meat; fish; raw vegetables; potatoes and cooked vegetables; eggs; sandwiches; cold cuts and appetizers; cheeses; fruit; bread and wine; coffee, milk and pastries; flavors and spices; and cooking methods. Each food was converted into mean daily intake in grams, and the total was summarized in 33 food groups (Supplementary Table S1) established according to similarity type [37,38].

### 2.3. Statistical Analysis

The subjects characteristics are reported as median and interquartile range (IQR) for continuous variables, and as frequencies and percentages (%) for categorical variables. The distribution of allelic frequencies in the MASLD or no MASLD sub-cohorts was studied, and deviations from Hardy–Weinger equilibrium (HWE) were calculated. Furthermore, the Minor Allele Frequency (MAF) was calculated.

The Kruskal–Wallis rank test was used to evaluate the variations in food group intake across genotypes groups, and Dunn’s post-hoc test was performed for multiple pairwise comparisons, with Bonferroni adjustments. We estimated a multiple logistic regression model using MASLD (yes vs. no) at NUTRIHEP 2 as the outcome variable and food group intake at NUTRIHEP 1 as the predictors to evaluate the relationship between food intake at the first visit and the development of liver disease by the time of the NUTRIHEP recall. The models were also adjusted for some covariates at the different times to underlie the predictive role of variables on the dependent variable (gender, age at NUTRIHEP 1, BMI at NUTRIHEP 2, and kcal intake at NUTRIHEP 1), then the estimated coefficients were transformed into Odds Ratios (ORs) and relative confidence intervals at 95%.

To test the null hypothesis of non-association, the two-tailed probability level was set at 0.05. The Pearson or Hosmer–Lemeshow test was used to evaluate the goodness-of-fit for the logistic regression models built [39]. To summarize and visualize the information about food group intake and to create a profile of patients by genotype and steatosis, a principal component analysis (PCA) was performed. This was useful to extract information from the data matrix and reduce it to fit new groups of variables. The analyses were conducted using StataCorp 2023 Stata Statistical Software: Release 18 (College Station, TX, USA: StataCorp LLC.), while RStudio (“Chocolate Cosmos” Release 2024.04.0) was used for the plots.

## 3. Results

### 3.1. Patient Characteristics

The sociodemographic and clinical characteristics of the total cohort and subcohorts stratified by genotype and recalled for a first follow-up [23] are reported in Table 1 and Table 2, considering the baseline characteristics of the covariates included in the association models.

**Figure 1:**
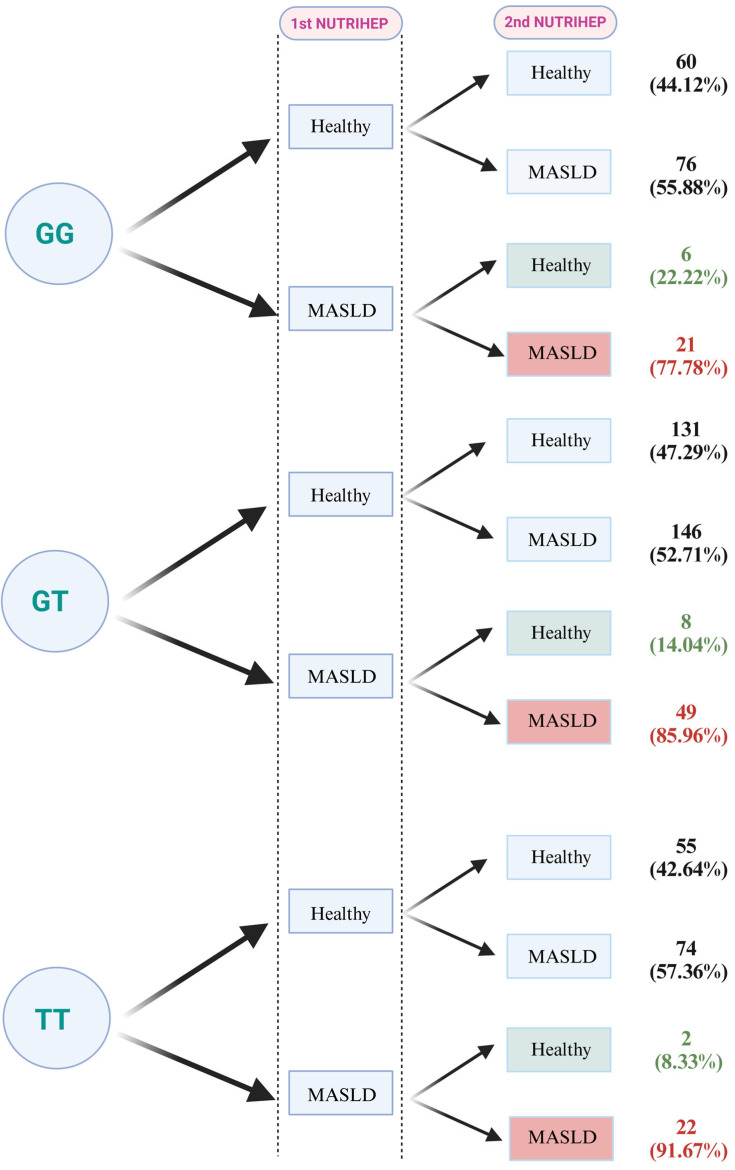
Evolution of MASLD disease over the study period, stratified by genotype. The disease condition is shown in red, while healthy subjects are highlighted in green (Created in https://BioRender.com).

The choice to consider the disease only in NUTRIHEP 2 was based on its pathological characteristics, which are completely reversible and strongly influenced by lifestyle. To confirm this, we evaluated the subjects both at baseline (NUTRIHEP 1) and at the first follow-up (NUTRIHEP 2). For subjects in the FOXO3 GG category, it emerged that 60 (44.12%) were healthy at the first and second visit, 76 (55.88%) developed MASLD, 6 (22.22%) regressed from MASLD to a healthy condition, and 21 (77.78%) remained affected. As regards the GT category, instead, 131 (47.29%) were healthy at the first and second visit, 146 (52.71%) developed the disease, 8 (14.04%) regressed from MASLD to a healthy condition, and 49 (85.96%) remained affected. Finally, in the TT homozygotes, 55 (42.64%) were healthy at the first and second visit, 74 (57.36%) developed MASLD, 2 (8.33%) regressed from MASLD to a healthy condition, and 22 (91.67%) remained affected (Figure 1).

### 3.2. The Impact of rs2802292 SNP on Food Intake in a Southern Italian Cohort

To characterize how gene–diet interactions influence hepatic steatosis, we evaluated the effects of individual food types across genetic strata. In this analysis, we identified numerous associations between dietary intake and genotypes. The daily consumption of the food groups at NUTRIHEP 1 stratified by GG, GT, and TT, is shown in Table 3.

No intake differences were found between genotypes, except for added sugars and processed meat. Specifically, the TT subjects consumed more added sugars than the GT or GG subjects (8.00 (16.00), 8.05 (15.80), and 4.35 (12.10), *p* = 0.02), showing the same trend for processed meat (9.90 (16.65), 9.15 (11.80), and 5.25 (8.10), *p* = 0.01), which was statistically significant. Borderline results were found for seafood/shellfish (7.50 (19.90), 4.70 (9.70), and 6.95 (12.10), *p* = 0.09). Conversely, the GG subjects had a higher fruit intake compared to other groups (275.00 (178.90), 286.10 (214.60), 315.95 (228.80), *p* = 0.07). Despite differences in grams/daily of specific foods consumed, the total calorie intake remained the same (*p* = 0.10), underlining how combinations of foods, although different between genotypes, actually lead to the same total nutritional intake. Comparative analysis allowed us to evaluate only the differences between the groups, but not the relationship between parameters and outcomes. For this reason, to evaluate the relationship among dietary intake, MASLD, and the genotypes, multivariate logistic models were built, adjusted for confounding variables strictly associated with the outcome of interest.

### 3.3. Primary Outcome: Logistic Models and Food Group Effects on MASLD

The association between food intake and MASLD was analyzed, as shown in Table 4.

In this analysis, we identified different associations in the subcohorts. Other dressing fats resulted in protection against MASLD in GG subjects (OR = 0.953, *p* = 0.038, 0.910 to 0.997 95% C.I.), while seed oil consumption and juices had the same protective role in the other genotypes, i.e., GT (OR = 0.687, *p* = 0.047, 0.474 to 0.996 95% C.I., OR = 0.993, *p* = 0.002, 0.988 to 0.997, 95% C.I., respectively). Conversely, the risk of MASLD was enhanced by spirits intake (OR = 1.050, *p* = 0.005, 1.014 to 1.086, 95% C.I.). In the TT subjects, grains had a protective role (OR = 0.991, *p* = 0.012, 0.984 to 0.998, 95% C.I.), whereas white meat (OR = 1.018, *p* = 0.031, 1.002 to 1.035, 95% C.I.), ready-to-eat dishes (OR = 1.015, *p* = 0.033, 1.001 to 1.029, 95% C.I.), processed meat (OR = 1.093, *p* = 0.003, 1.031 to 1.158, 95% C.I.), and processed fish (OR = 1.085, *p* = 0.037, 1.005 to 1.172 95% C.I.) were risk factors for MASLD development in this genotype category.

Disease conditions based on absolute frequencies in the genotype categories are shown in Figure S1. Changes in MASLD categories over time show how different subjects evolved during the period.

To summarize and visualize the information about food groups intake and create a profile of subjects by MASLD condition, PCA was performed. Supplementary Figure S2 shows a biplot (Supplementary Figure S2A,B) in which the variable coordinates are represented as projections inside the circle (i.e., dimension 1 × dimension 2 plane). This biplot shows no clear separation between genotypes, and no clear identification of a distinct MASLD-specific condition in the three genotypes analyzed. In the first two dimensions (Supplementary Figure S2A), the variance explained was 12.80% and 7.60%, respectively, while it was 13.10% and 8.60% for the subjects with MASLD (Supplementary Figure S2B).

The HWE was not significant (*p* = 0.51), indicating that there were no significant differences between observed and expected genotype frequencies, in both MASLD and non-MASLD subjects, suggesting that this cohort was in Hardy–Weinberg equilibrium and the HWE assumptions were met. Moreover, the MAF of FOXO3 rs2802292 (G-Allele) was 0.49 in this cohort (n = 650).

When considering the effect of food on MASLD, it is essential to assess both immediate (short-term) and long-term effects, but focusing on the long-term impact tends to be more useful for overall liver health. Short-term assessment helps to understand immediate metabolic responses to dietary changes, as certain foods can cause sharp spikes or drops in liver fat deposition. However, the main limitation is that the liver can temporarily adapt to these changes, so short-term assessments may not capture the lasting consequences of an unhealthy diet. In contrast, long-term assessment allowed assessment of a progressive state of liver disease, and this has been linked to chronic dietary patterns. Therefore, studies similar to ours are critical in understanding the sustained impact of certain foods on liver fat accumulation, inflammation, and possibly fibrosis. Although the consumption of some foods changed over time, obviously due to the aging of the cohort, as well as to changes in dietary habits, and probably due to age-associated physiological changes (including slower gastric emptying, altered hormonal responses, a reduced basal metabolism, and altered sense of taste and smell) [40], calorie requirements (kcal) remained unchanged in each genotype (Table 5, *p* > 0.05).

Furthermore, to evaluate the immediate food consumption habits at the first follow-up, association analysis was performed, building models adjusted for the same covariates adopted for long-time analysis. A non-protective role of fruiting vegetables was found in the GG group (OR = 1.012, *p* = 0.048, 1.000 to 1.024, 95% C.I.), while in the GT genotype, the risk effect was found for leafy vegetables (OR = 1.014, *p* = 0.036, 1.001 to 1.026, 95% C.I.), root vegetables (OR = 1.017, *p* = 0.033, 1.001 to 1.033, 95% C.I.), and seed oil (OR = 1.119, *p* = 0.034, 1.009 to 1.243, 95% C.I.). In TT subjects, other vegetables (OR = 1.027, *p* = 0.019, 1.004 to 1.050, 95% C.I.), juices (OR = 1.010, *p* = 0.045, 1.000 to 1.019, 95% C.I.), and ready-to-eat dishes (OR = 1.026, *p* = 0.028, 1.003 to 1.050, 95% C.I.) had a risk role on MASLD. Furthermore, these foods confirm their short-term effect on liver health, although this was characterized by a decrease in the recall compared to the baseline.

## 4. Discussion

Our examination of associations between the consumption of certain food groups and the risk of developing MASLD in the sub-cohort of genotypes yielded some interesting results. Based on our studies, GG subjects consuming 1 incremental unit of other dressing fat can even have a 4% reduced risk of developing MASLD. Similarly, GT subjects consuming juices and seed oil have a 1% and 31% reduction in the risk of developing the disease, respectively, compared to the 4% increased risk of developing it caused by consuming spirits. The interest of this cohort is focused on subjects with a TT profile. In these subjects, with the exception of grains, the intake of processed fish and meat, ready-to-eat dishes and white meat represents a risk for the development of the disease. These findings highlight how these profiles are classified at “risk” when they consume foods classifiable as “negative”, with pro-inflammatory effects, unlike those with at least one G allele who are protected even when consuming these foods.

The list of foods that show adverse effects for the development of MASLD has been widely proven to be harmful for health. MASLD is a condition characterized by the accumulation of excess fat in the liver, not due to alcohol consumption. Indeed, recent studies have shown associations between certain types of foods, including processed meats, ready-to-eat dishes, and white meat, and the risk of developing MASLD.

Processed meats are rich in saturated fats, preservatives, and additives such as nitrites, which can contribute to oxidative stress and inflammation in the liver. These factors are key contributors to fat accumulation in the liver and the progression of liver disease [41,42]. In addition to saturated fat, major contributors include heme iron and specific cooking methods, such as advanced glycation end products (AGEs), heterocyclic amines, and other byproducts of muscle protein oxidation [43]. Diets high in processed meats often increase the overall calorie intake and induce poor metabolic health, which are both risk factors for MASLD.

Ready-to-eat dishes are often highly processed, calorie-dense, and have a rich content of unhealthy fats and sugars. They tend to be low in fiber and essential nutrients. Frequent consumption of these dishes is linked to weight gain, metabolic syndrome, and insulin resistance, all of which are risk factors for MASLD [44].

As for meat, the effects of white meat on MASLD are less clear compared to those of processed meats. White meat, when cooked using a healthy method, is generally considered leaner and lower in saturated fat. Studies suggest that lean white meat may be a healthier protein source compared to red and processed meats [45]. A balanced diet rich in fruits, vegetables, whole grains, and lean proteins, along with physical activity, can reduce the risk of MASLD and improve liver health, especially in those subjects with a constitutive FOXO3 TT genetic profile.

Indeed, one of the most important challenges in the field of MASLD is the identification and management of patients affected by this metabolic disease. Until now, the medical treatment of MASLD has focused on weight reduction, mainly through dietary intervention [46]. Instead, our results highlight that having a certain genotype could be a risk factor for the progression or worsening of the disease, independently of dietary habits.

### Strengths and Limitations

Although this study has the strength of including a large cohort from a specific Italian geographical area and represents a novel contribution to the field of dietary habits and clinical outcomes that could depend on having a certain genotype, it nevertheless has some limitations. In the future, it would be useful to include other cohorts from the same geographical area to verify the allelic distribution and the current overlap in dietary habits, in order to confirm these findings, which have not yet been explored in depth. Furthermore, it would be important to demonstrate in the laboratory, using mouse models, how this SNP could contribute to the evolution of liver disease by acting on certain metabolic pathways.

In addition, one of the limitations of this study could be the possible underestimation or overestimation of the data [47] collected through the food questionnaire. These biases could derive from memory errors, the desire to provide socially acceptable answers, or from a wrong perception of one’s eating habits. These biases could partly influence the accuracy of the observed associations, making it necessary to integrate them with more objective assessment methods, such as the use of nutritional biomarkers or direct food monitoring, although dietary questionnaire are currently one of the simplest and cheapest methods for assessing eating habits.

Furthermore, although this study has produced important results, it is limited by the evaluation of the interaction between a single functional SNP rs2802292, diet, and risk of MASLD. In fact, so far, genome-wide [48,49] association studies have identified several genetic variants associated with MASLD. Investigating the combined effect of these genetic variants with long-term follow-up data of participants in future research could provide a greater understanding of how genetic factors and dietary habits influence the risk of MASLD.

## 5. Conclusions

The FOXO3 rs2802292 G allele may play a protective role in MASLD subjects in relation to certain food intake patterns, influencing their clinical outcomes. In particular, understanding the mechanisms and possible secondary effects on liver disease could provide insights into personalized nutritional strategies aimed at mitigating disease progression and promoting healthier eating habits

## Figures and Tables

**Table 1:** Sociodemographic parameters of subjects included in this study. Nutrihep 1 and 2 cohorts.

Parameters *	Cohorts					
	Nutrihep 1 (2005–2006) (*n* = 650)			Nutrihep 2 (2014–2016) (*n* = 650)		
	GG (*n* = 163)	GT (*n* = 334)	TT (*n* = 153)	GG (*n* = 163)	GT (*n* = 334)	TT (*n* = 153)
Gender (M) (%)	65 (39.88)	142 (42.51)	67 (43.79)	65 (39.88)	142 (42.51)	67 (43.79)
Age (yrs)	52.00 (16.00)	50.00 (16.00)	53.00 (14.00)	62.00 (16.00)	61.00 (16.00)	64.00 (15.00)
Age Classes (%)						
Adults (≤65)	141 (86.50)	307 (91.92)	140 (91.50)	98 (60.12)	210 (62.87)	86 (56.21)
Elderly (>65)	22 (13.50)	27 (8.08)	13 (8.50)	65 (39.88)	124 (37.13)	67 (43.79)
Education (%)						
None	7 (4.43)	6 (1.88)	6 (4.14)	4 (2.60)	4 (1.25)	6 (4.08)
Elementary School	48 (30.38)	100 (31.35)	50 (34.48)	44 (28.57)	99 (31.03)	50 (34.01)
Secondary School	51 (32.28)	111 (34.80)	57 (39.31)	51 (33.12)	101 (31.66)	52 (35.37)
High School	47 (29.75)	81 (25.39)	27 (18.62)	43 (27.92)	90 (28.21)	30 (20.41)
Degree	5 (3.16)	21 (6.58)	5 (3.45)	7 (4.55)	15 (4.70)	0 (0.00)
Post-Degree	0 (0.00)	0 (0.00)	0 (0.00)	5 (3.25)	10 (3.13)	9 (6.12)
Civil Status (%)						
Single	20 (12.58)	33 (10.38)	19 (13.10)	14 (9.09)	26 (8.15)	8 (5.44)
Married or Cohabiting	131 (82.39)	265 (83.33)	120 (82.76)	126 (81.82)	257 (80.56)	125 (85.03)
Divorced or Separated	4 (2.52)	9 (2.83)	1 (0.69)	3 (1.95)	12 (3.76)	2 (1.36)
Widow/er	4 (2.52)	11 (3.46)	5 (3.45)	11 (7.14)	24 (7.52)	12 (8.16)
Smoker (Yes) (%)	24 (15.29)	45 (14.15)	23 (15.86)	18 (11.46)	37 (11.56)	16 (10.96)

* As median and interquartile range (IQR) for continuous variables, and as frequency and percentage (%) for categorical variables.

**Table 2:** Clinical parameters of subjects included in this study. Nutrihep 1 and 2 cohorts.

Parameters *	Cohorts					
	Nutrihep 1 (2005–2006) (*n* = 650)			Nutrihep 2 (2014–2016) (*n* = 650)		
	GG (*n* = 163)	GT (*n* = 334)	TT (*n* = 153)	GG (*n* = 163)	GT (*n* = 334)	TT (*n* = 153)
BMI (kg/m^2^)	25.97 (5.88)	25.62 (5.57)	26.23 (4.48)	27.69 (6.55)	27.50 (6.53)	27.80 (6.63)
Systolic Blood Pressure (mmHg)	125.00 (15.00)	125.00 (15.00)	125.00 (15.00)	120.00 (30.00)	120.00 (20.00)	130.00 (30.00)
Diastolic Blood Pressure (mmHg)	80.00 (10.00)	80.00 (10.00)	80.00 (5.00)	80.00 (10.00)	80.00 (10.00)	80.00 (10.00)
Diabetes (Yes) (%)	5 (3.47)	10 (3.39)	7 (5.19)	13 (7.98)	23 (6.89)	22 (14.38)
Hypertension (Yes) (%)	35 (24.31)	61 (20.68)	29 (21.48)	63 (40.38)	134 (42.14)	64 (44.14)
MASLD (Yes) (%)	27 (16.56)	57 (17.07)	24 (15.69)	97 (59.51)	195 (58.38)	96 (62.75)

* As median and interquartile range (IQR) for continuous variables, and as frequency and percentage (%) for categorical variables. Abbreviations: BMI, body mass index; MASLD, Metabolic Dysfunction-Associated Steatotic Liver Disease.

**Table 3:** Dietary daily intakes of 33 food groups in the total cohort and in subjects stratified by genotype at NUTRIHEP 1 (*n* = 650).

Food-Groups *	Total Cohort	Genotypes			*p* ^^^
		GG	GT	TT	
Dairy	79.20 (130.30)	71.60 (119.30)	80.5 (133.60)	80.20 (123.90)	0.26
Low Fat Dairy	91.10 (205.30)	90.30 (204.90)	120.0 (204.90)	51.40 (206.10)	0.52
Eggs	3.50 (6.90)	3.50 (7.00)	3.50 (6.90)	3.50 (5.80)	0.48
White Meat	35.40 (31.40)	33.50 (29.30)	35.35 (30.70)	37.20 (5.80)	0.43
Red Meat	45.60 (37.50)	42.75 (36.30)	46.0 (36.90)	51.50 (44.00)	0.25
Fish	16.20 (17.30)	16.20 (17.20)	16.20 (17.20)	16.20 (17.20)	0.60
Seafood/Shellfish	5.50 (10.40)	6.95 (12.10)	4.70 (9.70)	7.50 (19.90)	0.09
Leafy Vegetables	18.50 (21.40)	17.50 (26.60)	18.80 (20.70)	17.50 (19.90)	0.86
Fruiting Vegetables	45.80 (54.20)	49.10 (57.40)	45.10 (51.50)	46.00 (51.50)	0.58
Root Vegetables	11.30 (18.50)	11.35 (21.60)	11.05 (18.80)	11.40 (15.70)	0.91
Other Vegetables	44.50 (40.40)	46.35 (42.90)	44.10 (40.20)	43.90 (36.80)	0.75
Herbs and Spices	0.30 (2.00)	0.30 (2.00)	0.30 (2.00)	0.80 (3.00)	0.55
Legumes	37.30 (35.00)	37.25 (27.60)	38.70 (37.30)	33.90 (36.30)	0.33
Potatoes	16.00 (17.60)	16.10 (17.60)	12.10 (17.60)	16.10 (15.00)	0.52
Fruits	290.80 (210.50)	315.95 (228.80)	286.10 (214.60)	275.00 (178.90)	0.07
Nuts	0.40 (1.70)	0.40 (2.30)	0.40 (1.70)	0.60 (1.70)	0.84
Grains	109.00 (114.70)	97.60 (89.10)	110.90 (122.90)	109.90 (111.20)	0.21
Olive Oil	20.60 (13.40)	20.40 (13.10)	20.60 (13.40)	21.20 (14.20)	0.84
Seed Oil	0.00 (0.00)	0.00 (0.00)	0.00 (0.00)	0.00 (0.00)	0.16
Other Dressing Fats	2.00 (6.00)	1.85 (4.60)	1.90 (6.70)	2.80 (6.30)	0.10
Sweets	68.20 (69.70)	69.20 (63.70)	69.65 (72.40)	63.90 (55.60)	0.19
Added Sugars	8.00 (16.00)	4.35 (12.10)	8.05 (15.80)	8.00 (16.00)	0.02 ^a^
Juices	16.60 (71.40)	8.40 (71.40)	16.60 (71.40)	16.60 (71.40)	0.54
Caloric Drinks	0.00 (26.70)	0.00 (26.70)	0.00 (26.70)	0.00 (20.00)	0.82
Ready to Eat Dish	49.00 (39.90)	46.25 (33.60)	50.85 (49.50)	47.60 (39.20)	0.14
Coffee and Tea	60.00 (60.00)	60.00 (60.00)	60.00 (60.00)	60.00 (60.00)	0.99
Wine	35.70 (125.00)	17.90 (125.00)	35.70 (125.00)	53.60 (142.80)	0.17
Beer	5.50 (44.00)	5.50 (33.00)	5.50 (47.10)	5.50 (33.00)	0.98
Spirits	2.00 (4.60)	2.00 (3.30)	2.00 (3.30)	2.00 (6.00)	0.10
Processed Foods					
Eggs	4.70 (10.20)	5.25 (10.20)	4.70 (10.20)	5.80 (9.30)	0.94
Meat	7.70 (12.10)	5.25 (8.10)	9.15 (11.80)	9.90 (16.60)	0.01 ^b^
Fish	6.30 (9.60)	5.90 (10.80)	6.50 (10.20)	6.30 (7.20)	0.62
Grains	23.80 (44.20)	20.00 (36.20)	23.50 (51.10)	27.00 (47.60)	0.10
Kcal	1865.57 (909.32)	1824.34 (702.93)	1919.34 (1036.42)	1913.14 (935.24)	0.10

* As median and interquartile range (IQR). Food groups were calculated on the quantity of daily consumption (grams). ^^^ Kruskal–Wallis rank test. Dunn’s post-hoc test: ^a^ GT vs. GG (*p* = 0.009); ^b^ GT vs. GG (*p* = 0.02); TT vs. GG (*p* = 0.01).

**Table 4:** Logistic regression model of MASLD at NUTRIHEP 2 (yes vs. no) on food group intake at NUTRIHEP 1, stratified for genotypes.

Food-Group	GG				GT				TT			
	OR	se (OR)	*p*	95% C.I.	OR	se (OR)	*p*	95% C.I.	OR	se (OR)	*p*	95% C.I.
Dairy	0.998	0.002	0.354	0.994 to 1.002	0.999	0.001	0.444	0.996 to 1.001	0.997	0.002	0.143	0.993 to 1.001
Low Fat Dairy	1.002	0.001	0.07	0.999 to 1.005	0.999	0.001	0.683	0.998 to 1.001	1.002	0.002	0.338	0.998 to 1.005
Eggs	1.051	0.035	0.130	0.985 to 1.122	1.003	0.023	0.889	0.959 to 1.048	1.027	0.037	0.452	0.957 to 1.102
White Meat	1.005	0.009	0.618	0.986 to 1.023	1.002	0.006	0.680	0.991 to 1.014	1.018	0.009	0.031	1.002 to 1.035
Red Meat	0.992	0.007	0.214	0.979 to 1.005	1.006	0.004	0.102	0.999 to 1.014	0.986	0.007	0.062	0.972 to 1.001
Fish	1.005	0.014	0.724	0.977 to 1.033	0.986	0.008	0.098	0.970 to 1.002	1.000	0.012	0.967	0.977 to 1.025
Seafood/Shellfish	0.984	0.026	0.552	0.934 to 1.037	0.981	0.016	0.269	0.949 to 1.014	1.038	0.03	0.140	0.988 to 1.091
Leafy Vegetables	1.007	0.007	0.365	0.992 to 1.021	1.001	0.006	0.884	0.989 to 1.012	0.999	0.008	0.871	0.983 to 1.014
Fruiting Vegetables	1.006	0.004	0.137	0.998 to 1.014	0.995	0.003	0.178	0.989 to 1.002	0.999	0.004	0.928	0.991 to 1.008
Root Vegetables	0.989	0.010	0.308	0.969 to 1.010	0.997	0.006	0.604	0.984 to 1.009	0.999	0.014	0.948	0.971 to 1.027
Other Vegetables	0.996	0.005	0.515	0.986 to 1.007	1.001	0.004	0.675	0.994 to 1.009	0.999	0.007	0.945	0.985 to 1.014
Herbs and Spices	1.089	0.634	0.142	0.972 to 1.221	0.972	0.040	0.493	0.896 to 1.054	1.007	0.051	0.883	0.912 to 1.113
Legumes	0.996	0.006	0.531	0.984 to 1.008	0.999	0.003	0.823	0.993 to 1.005	1.014	0.008	0.083	0.998 to 1.030
Potatoes	0.997	0.012	0.834	0.974 to 1.021	0.994	0.007	0.409	0.979 to 1.009	1.014	0.014	0.300	0.987 to 1.042
Fruits	0.999	0.001	0.294	0.996 to 1.001	1.000	0.001	0.983	0.999 to 1.001	0.998	0.001	0.214	0.996 to 1.001
Nuts	0.928	0.041	0.097	0.850 to 1.014	0.983	0.032	0.600	0.923 to 1.047	1.073	0.120	0.527	0.862 to 1.335
Grains	1.001	0.003	0.790	0.995 to 1.007	0.998	0.002	0.348	0.994 to 1.002	0.991	0.003	0.012	0.984 to 0.998
Olive Oil	1.037	0.023	0.107	0.992 to 1.084	0.997	0.014	0.843	0.969 to 1.026	1.015	0.026	0.569	0.965 to 1.067
Seed Oil	--	--	--	--	0.687	0.130	0.047	0.474 to 0.996	0.618	0.634	0.639	0.083 to 4.619
Other Dressing Fats	0.953	0.022	0.038	0.910 to 0.997	1.006	0.010	0.558	0.987 to 1.025	1.018	0.027	0.500	0.966 to 1.073
Sweets	1.004	0.004	0.359	0.995 to 1.013	0.998	0.002	0.582	0.994 to 1.003	1.004	0.004	0.320	0.996 to 1.011
Added Sugars	1.006	0.019	0.763	0.969 to 1.044	0.981	0.014	0.181	0.954 to 1.009	0.996	0.012	0.766	0.973 to 1.020
Juices	0.997	0.003	0.290	0.992 to 1.002	0.993	0.002	0.002	0.988 to 0.997	0.999	0.002	0.997	0.995 to 1.004
Caloric Drinks	0.996	0.004	0.398	0.988 to 1.004	1.000	0.002	0.879	0.996 to 1.003	1.000	0.004	0.988	0.992 to 1.008
Ready to Eat Dish	0.992	0.007	0.290	0.978 to 1.007	1.004	0.003	0.149	0.998 to 1.012	1.015	0.007	0.033	1.001 to 1.029
Coffee and Tea	1.001	0.002	0.453	0.998 to 1.004	0.998	0.001	0.285	0.995 to 1.001	1.001	0.002	0.732	0.997 to 1.004
Wine	1.000	0.002	0.956	0.996 to 1.004	1.002	0.001	0.159	0.999 to 1.004	1.001	0.002	0.662	0.997 to 1.004
Beer	0.998	0.002	0.271	0.993 to 1.002	1.001	0.001	0.539	0.998 to 1.004	1.006	0.005	0.222	0.996 to 1.015
Spirits	0.996	0.008	0.667	0.981 to 1.013	1.050	0.018	0.005	1.014 to 1.086	1.028	0.002	0.198	0.985 to 1.073
Processed Foods												
Eggs	1.014	0.023	0.543	0.970 to 1.060	0.988	0.016	0.446	0.958 to 1.019	0.990	0.020	0.638	0.951 to 1.031
Meat	1.022	0.023	0.316	0.979 to 1.068	1.015	0.012	0.196	0.992 to 1.038	1.093	0.032	0.003	1.031 to 1.158
Fish	0.995	0.021	0.807	0.954 to 1.040	0.983	0.015	0.206	0.956 to 1.010	1.085	0.042	0.037	1.005 to 1.172
Grains	0.999	0.006	0.998	0.990 to 1.011	1.002	0.003	0.453	0.996 to 1.009	0.999	0.005	0.873	0.989 to 1.009

Abbreviations: OR, Odds Ratio; se (OR), Standard Error of OR; 95% C.I., Confidential Interval at 95%. Model adjusted for age in NUTRIHEP 2, gender, BMI at NUTRIHEP 2, and Kcal in NUTRIHEP 1.

**Table 5:** Dietary daily intakes of 33 food groups between two follow-up calls.

Food-Groups *	Genotypes								
	GG		*p* ^^^	GT		*p* ^^^	TT		*p* ^^^
	NUTRIHEP 1	NUTRIHEP 2		NUTRIHEP 1	NUTRIHEP 2		NUTRIHEP 1	NUTRIHEP 2	
Dairy	71.60 (119.30)	44.90 (95.70)	0.02	80.55 (133.60)	48.20 (89.20)	0.002	80.20 (123.90)	42.80 (99.55)	0.02
Low Fat Dairy	90.30 (204.90)	37.90 (197.50)	0.30	120.00 (204.90)	82.60 (200.20)	0.67	51.40 (206.10)	40.20 (135.75)	0.93
Eggs	3.50 (7.00)	7.10 (11.60)	0.0006	3.50 (6.90)	5.30 (7.00)	<0.0001	3.50 (5.80)	7.10 (13.30)	0.15
White Meat	33.50 (29.30)	19.90 (27.40)	<0.0001	35.35 (30.70)	17.05 (24.50)	<0.0001	37.20 (36.30)	17.65 (27.95)	<0.0001
Red Meat	42.75 (36.30)	33.80 (33.90)	0.13	46.00 (36.90)	34.30 (33.00)	<0.0001	51.50 (44.00)	39.80 (36.90)	0.0006
Fish	16.20 (17.29)	11.60 (15.90)	0.0006	16.20 (17.20)	11.60 (13.20)	0.0004	16.20 (17.20)	12.60 (16.40)	0.06
Seafood/Shellfish	6.95 (12.10)	5.10 (12.10)	0.79	4.70 (9.70)	4.50 (10.70)	0.76	7.50 (10.80)	7.00 (12.35)	0.43
Leafy Vegetables	17.50 (26.60)	24.20 (33.20)	0.09	18.80 (20.70)	19.20 (27.90)	0.19	17.50 (19.90)	17.45 (21.35)	0.93
Fruiting Vegetables	49.10 (57.40)	48.80 (51.30)	0.87	45.10 (51.50)	39.25 (50.00)	0.21	46.00 (51.50)	43.95 (52.60)	0.93
Root Vegetables	11.35 (21.60)	11.00 (17.40)	0.55	11.05 (18.80)	11.65 (15.90)	0.99	11.40 (15.70)	9.40 (17.50)	0.38
Other Vegetables	46.35 (42.90)	28.10 (31.40)	<0.0001	44.10 (40.20)	29.25 (25.00)	<0.0001	43.90 (36.80)	27.00 (24.50)	<0.0001
Herbs and Spices	0.30 (2.00)	0.00 (1.30)	0.0006	0.30 (2.00)	0.00 (1.10)	<0.0001	0.80 (3.00)	0.00 (1.30)	<0.0001
Legumes	37.25 (27.60)	18.20 (26.10)	<0.0001	38.70 (37.30)	17.95 (22.90)	<0.0001	33.90 (36.30)	18.30 (33.55)	0.0003
Potatoes	16.10 (17.60)	21.30 (12.30)	0.61	12.10 (17.60)	21.40 (12.30)	0.10	16.10 (15.00)	16.00 (16.95)	0.29
Fruits	315.95 (228.80)	347.40 (266.40)	0.01	286.10 (214.60)	341.30 (224.20)	0.01	275.00 (178.90)	337.75 (199.20)	0.02
Nuts	0.40 (2.30)	8.80 (22.90)	<0.0001	0.40 (1.70)	5.90 (16.80)	<0.0001	0.60 (1.70)	8.30 (19.35)	<0.0001
Grains	97.60 (89.10)	72.40 (94.60)	0.0002	110.90 (122.90)	66.20 (89.10)	<0.0001	109.90 (111.20)	79.80 (97.00)	<0.0001
Olive Oil	20.40 (13.10)	1.30 (2.90)	<0.0001	20.60 (13.40)	1.20 (3.00)	<0.0001	21.10 (14.20)	1.65 (2.85)	<0.0001
Seed Oil	0.00 (0.00)	1.80 (3.80)	<0.0001	0.0 (0.00)	1.90 (3.90)	<0.0001	0.00 (0.00)	1.80 (4.00)	<0.0001
Other Dressing Fats	1.85 (4.60)	1.00 (2.50)	0.004	1.90 (6.70)	1.10 (2.90)	<0.0001	2.80 (6.30)	1.20 (2.95)	<0.0001
Sweets	69.20 (63.70)	59.80 (66.60)	0.61	69.65 (72.40)	67.15 (57.70)	0.01	63.90 (55.60)	51.30 (44.55)	0.13
Added Sugars	4.35 (12.10)	8.00 (11.40)	0.78	8.05 (15.80)	8.0 (11.90)	0.28	8.00 (16.00)	6.15 (10.50)	0.35
Juices	16.60 (71.40)	0.00 (41.70)	0.002	16.60 (71.40)	0.00 (41.60)	<0.0001	16.60 (71.40)	0.00 (25.00)	0.003
Caloric Drinks	0.0 (26.70)	0.00 (13.30)	0.02	0.00 (26.70)	0.00 (6.70)	<0.0001	0.00 (20.00)	0.00 (6.70)	0.0001
Ready to Eat Dish	46.25 (33.60)	34.00 (31.60)	<0.0001	50.85 (49.50)	39.85 (33.10)	<0.0001	47.60 (39.20)	38.30 (29.95)	<0.0001
Coffee and Tea	60.00 (60.00)	81.40 (120.00)	0.004	60.00 (60.00)	81.40 (107.10)	<0.0001	60.00 (60.00)	73.95 (98.60)	0.03
Wine	17.90 (125.00)	16.60 (125.00)	0.67	35.70 (125.00)	17.90 (125.00)	0.94	53.60 (142.80)	26.80 (125.00)	0.03
Beer	5.50 (33.00)	5.50 (44.00)	0.99	5.50 (47.10)	5.50 (47.10)	0.99	5.50 (33.00)	5.50 (22.00)	0.07
Spirits	2.00 (3.30)	1.30 (4.00)	0.0005	2.00 (3.30)	1.30 (5.30)	0.0008	2.00 (6.00)	1.30 (2.35)	0.0001
Processed Foods									
Eggs	5.25 (10.20)	9.30 (14.00)	0.005	4.70 (10.20)	9.30 (15.30)	0.0006	5.80 (9.30)	9.30 (9.30)	0.99
Meat	5.25 (8.10)	7.90 (8.80)	0.06	9.15 (11.80)	7.70 (12.60)	0.95	9.90 (16.60)	7.25 (10.30)	0.07
Fish	5.90 (10.80)	9.0 (9.50)	0.02	6.40 (10.20)	7.60 (9.50)	0.02	6.30 (7.20)	7.60 (7.95)	0.25
Grains	20.00 (36.20)	16.30 (34.50)	0.30	23.50 (51.10)	22.20 (33.30)	<0.0001	27.00 (47.60)	18.75 (42.05)	0.02
Kcal	1824.34 (702.93)	1934.01 (757.32)	0.09	1919.34 (1036.42)	1976.60 (998.57)	0.21	1913.14 (935.24)	1882.79 (785.96)	0.19

* As median and interquartile range (IQR). Food groups were defined based on the quantity of daily consumption (grams). ^^^ Wilcoxon matched-pairs signed-rank test.

## Data Availability

The original data presented in this study are openly available in FigShare at https://doi.org/10.6084/m9.figshare.28677308.v3.

## Supplementary Materials

The following supporting information can be downloaded at: https://www.mdpi.com/article/10.3390/nu17081315/s1, Table S1: Individual foods from the FFQ (EPIC) and their corresponding food groupings used for the analyses; Figure S1: Alluvial plot of MASLD condition in the genotype categories; Figure S2: Biplot of PCA on food group intake, showing the projection of the data set on the PC1 × PC2 plane, stratified for genotypes (GG, GT, and TT) in subjects without MASLD (A) and with MASLD (B).

## References

[1] Santos B.F., Grenho I., Martel P.J., Ferreira B.I., Link W. (2023). FOXO family isoforms. Cell Death Dis..

[2] Cao G., Lin M., Gu W., Su Z., Duan Y., Song W., Liu H., Zhang F. (2023). The rules and regulatory mechanisms of FOXO3 on inflammation, metabolism, cell death and aging in hosts. Life Sci..

[3] Pizzino G., Irrera N., Cucinotta M., Pallio G., Mannino F., Arcoraci V., Squadrito F., Altavilla D., Bitto A. (2017). Oxidative Stress: Harms and Benefits for Human Health. Oxid. Med. Cell. Longev..

[4] Yan L.J., Levine R.L., Sohal R.S. (1997). Oxidative damage during aging targets mitochondrial aconitase. Proc. Natl. Acad. Sci. USA.

[5] Forman H.J., Zhang H. (2021). Targeting oxidative stress in disease: Promise and limitations of antioxidant therapy. Nat. Rev. Drug Discov..

[6] Chang Z.S., Xia J.B., Wu H.Y., Peng W.T., Jiang F.Q., Li J., Liang C.Q., Zhao H., Park K.S., Song G.H. (2019). Forkhead box O3 protects the heart against paraquat-induced aging-associated phenotypes by upregulating the expression of antioxidant enzymes. Aging Cell.

[7] Greer E.L., Oskoui P.R., Banko M.R., Maniar J.M., Gygi M.P., Gygi S.P., Brunet A. (2007). The energy sensor AMP-activated protein kinase directly regulates the mammalian FOXO3 transcription factor. J. Biol. Chem..

[8] Wang L., Zhu X., Sun X., Yang X., Chang X., Xia M., Lu Y., Xia P., Yan H., Bian H. (2019). FoxO3 regulates hepatic triglyceride metabolism via modulation of the expression of sterol regulatory-element binding protein 1c. Lipids Health Dis..

[9] Zhang K., Li L., Qi Y., Zhu X., Gan B., DePinho R.A., Averitt T., Guo S. (2012). Hepatic suppression of Foxo1 and Foxo3 causes hypoglycemia and hyperlipidemia in mice. Endocrinology.

[10] Shimokawa I., Komatsu T., Hayashi N., Kim S.E., Kawata T., Park S., Hayashi H., Yamaza H., Chiba T., Mori R. (2015). The life-extending effect of dietary restriction requires Foxo3 in mice. Aging Cell.

[11] Gree E.L., Brunet A. (2009). Different dietary restriction regimens extend lifespan by both independent and overlapping genetic pathways in C. elegans. Aging Cell.

[12] Brunet A., Sweeney L.B., Sturgill J.F., Chua K.F., Greer P.L., Lin Y., Tran H., Ross S.E., Mostoslavsky R., Cohen H.Y. (2004). Stress-dependent regulation of FOXO transcription factors by the SIRT1 deacetylase. Science.

[13] Neff F., Flores-Dominguez D., Ryan D.P., Horsh M., Schröder S., Adler T., Afonso L.C., Aguilar-Pimentel J.A., Becker L., Garrett L. (2013). Rapamycin extends murine lifespan but has limited effects on aging. J. Clin. Investig..

[14] Unnikrishnan A., Kurup K., Salmon A.B., Richardson A. (2020). Is Rapamycin a Dietary Restriction Mimetic?. J. Gerontol. A Biol. Sci. Med. Sci..

[15] Johnson S.C., Yanos M.E., Bitto A., Castanza A., Gagnidze A., Gonzalez B., Gupta K., Hui J., Jarvie C., Johnson B.M. (2015). Dose-dependent effects of mTOR inhibition on weight and mitochondrial disease in mice. Front. Genet..

[16] Eisenber T., Knauer H., Schauer A., Büttner S., Ruckenstuhl C., Carmona-Gutierrez D., Ring J., Schroeder S., Magnes C., Antonacci L. (2009). Induction of autophagy by spermidine promotes longevity. Nat. Cell Biol..

[17] Shintani T., Shintani H., Sato M., Ashida H. (2023). Calorie restriction mimetic drugs could favorably influence gut microbiota leading to lifespan extension. Geroscience.

[18] Willcox B.J., Donlon T.A., He Q., Chen R., Grove J.S., Yano K., Masaki K.H., Willcox D.C., Rodriguez B., Curb J.D. (2008). FOXO3A genotype is strongly associated with human longevity. Proc. Natl. Acad. Sci. USA.

[19] Flachsbart F., Dose J., Gentschew L., Geismann C., Caliebe A., Knecht C., Nygaard M., Badarinarayan N., ElSharawy A., May S. (2017). Identification and characterization of two functional variants in the human longevity gene FOXO3. Nat. Commun..

[20] Anselmi C.V., Malovini A., Roncarati R., Novelli V., Villa F., Condorelli G., Bellazzi R., Puca A.A. (2009). Association of the FOXO3A locus with extreme longevity in a southern Italian centenarian study. Rejuvenation Res..

[21] Bao J.M., Song X.L., Hong Y.Q., Zhu H.L., Zhang T., Chen W., Zhao S.C., Chen Q. (2014). Association between FOXO3A gene polymorphisms and human longevity: A meta-analysis. Asian J. Androl..

[22] Grossi V., Forte G., Sanese P., Peserico A., Tezil T., Lepore Signorile M., Fasano C., Lovaglio R., Bagnulo R., Loconte D.C. (2018). The longevity SNP rs2802292 uncovered: HSF1 activates stress-dependent expression of FOXO3 through an intronic enhancer. Nucleic Acids Res..

[23] Forte G., Donghia R., Lepore Signorile M., Tatoli R., Bonfiglio C., Losito F., De Marco K., Manghisi A., Guglielmi F.A., Disciglio V. (2024). Exploring the Relationship of rs2802292 with Diabetes and NAFLD in a Southern Italian Cohort-Nutrihep Study. Int. J. Mol. Sci..

[24] Maurice J., Manousou P. (2018). Non-alcoholic fatty liver disease. Clin. Med..

[25] Vanni E., Bugianesi E., Kotronen A., De Minicis S., Yki-Järvinen H., Svegliati-Baroni G. (2010). From the metabolic syndrome to NAFLD or vice versa?. Dig. Liver Dis..

[26] Friedman S.L., Neuschwander-Tetri B., Rinella M., Sanyal A.J. (2018). Mechanisms of NAFLD development and therapeutic strategies. Nat. Med..

[27] Marginean C.M., Pirscoveanu D., Cazacu S.M., Popescu M.S., Marginean I.C., Iacob G.A., Popescu M. (2024). Non-Alcoholic Fatty Liver Disease, Awareness of a Diagnostic Challenge—A Clinician’s Perspective. Gastroenterol. Insights.

[28] Targher G., Tilg H., Byrne C.D. (2021). Non-alcoholic fatty liver disease: A multisystem disease requiring a multidisciplinary and holistic approach. Lancet Gastroenterol. Hepatol..

[29] Huttasch M., Roden M., Kahl S. (2024). Obesity and MASLD: Is weight loss the (only) key to treat metabolic liver disease?. Metabolism.

[30] Younossi Z., Anstee Q.M., Marietti M., Hardy T., Henry L., Eslam M., George J., Bugianesi E. (2018). Global burden of NAFLD and NASH: Trends, predictions, risk factors and prevention. Nat. Rev. Gastroenterol. Hepatol..

[31] Riazi K., Azhari H., Charette J.H., Underwood F.E., King J.A., Afshar E.E., Swain M.G., Congly S.E., Kaplan G.G., Shaheen A.A. (2022). The prevalence and incidence of NAFLD worldwide: A systematic review and meta-analysis. Lancet Gastroenterol. Hepatol..

[32] Younossi Z.M., Golabi P., Paik J.M., Henry A., Van Dongen C., Henry L. (2023). The global epidemiology of nonalcoholic fatty liver disease (NAFLD) and nonalcoholic steatohepatitis (NASH): A systematic review. Hepatology.

[33] Donghia R., Bonfiglio C., Giannelli G., Tatoli R. (2025). Impact of educatin on Metabolic Dysfunction-Associated Steatotic Liver Disease (MASLD): A Southern Italy Cohort-Based study. J. Clin. Med..

[34] Tessari P., Coracina A., Cosma A., Tiengo A. (2009). Hepatic lipid metabolism and non-alcoholic fatty liver disease. Nutr. Metab. Cardiovasc. Dis..

[35] Cozzolongo R., Osella A.R., Elba S., Petruzzi J., Buongiorno G., Giannuzzi V., Leone G., Bonfiglio C., Lanzilotta E., Manghisi O.G. (2009). Epidemiology of HCV infection in the general population: A survey in a southern Italian town. Am. J. Gastroenterol..

[36] Rinella M.E., Lazarus J.V., Ratziu V., Francque S.M., Sanyal A.J., Kanwal F., Romero D., Abdelmalek M.F., Anstee Q.M., Arab J.P. (2023). NAFLD Nomenclature consensus group. A multisociety Delphi consensus statement on new fatty liver disease nomenclature. Hepatology.

[37] Shimotoyodome A., Suzuki J., Kameo Y., Hase T. (2011). Dietary supplementation with hydroxypropyl-distarch phosphate from waxy maize starch increases resting energy expenditure by lowering the postprandial glucose-dependent insulinotropic polypeptide response in human subjects. Br. J. Nutr..

[38] Donghia R., Pesole P.L., Coletta S., Bonfiglio C., De Pergola G., De Nucci S., Rinaldi R., Giannelli G. (2023). Food Network Analysis in Non-Obese Patients with or without Steatosis. Nutrients.

[39] Lemeshow S., Hosmer D.W.J. (1982). A review of goodness of fit statistics for use in the development of logistic regression models. Am. J. Epidemiol..

[40] Than N.N., Newsome P.N. (2015). Non-alcoholic fatty liver disease: When to intervene and with what. Clin. Med..

[41] Drewnowski A., Shultz J.M. (2001). Impact of aging on eating behaviors, food choices, nutrition, and health status. J. Nutr. Health Aging.

[42] Rahimi-Sakak F., Maroofi M., Emamat H., Hekmatdoost A. (2022). Red and Processed Meat Intake in Relation to Non-Alcoholic Fatty Liver Disease Risk: Results from a Case-Control Study. Clin. Nutr. Res..

[43] Ivancovsky-Wajcman D., Fliss-Isakov N., Grinshpan L.S., Salomone F., Lazarus J.V., Webb M., Shibolet O., Kariv R., Zelber-Sagi S. (2022). High Meat Consumption Is Prospectively Associated with the Risk of Non-Alcoholic Fatty Liver Disease and Presumed Significant Fibrosis. Nutrients.

[44] Henney A.E., Gillespie C.S., Alam U., Hydes T.J., Cuthbertson D.J. (2023). Ultra-Processed Food Intake Is Associated with Non-Alcoholic Fatty Liver Disease in Adults: A Systematic Review and Meta-Analysis. Nutrients.

[45] Hashemian M., Merat S., Poustchi H., Jafari E., Radmard A.R., Kamangar F., Freedman N., Hekmatdoost A., Sheikh M., Boffetta P. (2021). Red Meat Consumption and Risk of Nonalcoholic Fatty Liver Disease in a Population with Low Meat Consumption: The Golestan Cohort Study. Am. J. Gastroenterol..

[46] Perez-Diaz-Del-Campo N., Dileo E., Castelnuovo G., Nicolosi A., Guariglia M., Caviglia G.P., Rosso C., Armandi A., Bugianesi E. (2023). A nutrigenetic precision approach for the management of non-alcoholic fatty liver disease. Clin. Nutr..

[47] Khaled K., Hundley V., Bassil M., Bazzi M., Tsofliou F. (2021). Validation of the European Prospective Investigation into Cancer (EPIC) FFQ for use among adults in Lebanon. Public Health Nutr..

[48] Jamialahmadi O., De Vincentis A., Tavaglione F., Malvestiti F., Li-Gao R., Mancina R.M., Alvarez M., Gelev K., Maurotti S., Vespasiani-Gentilucci U. (2024). Partitioned polygenic risk scores identify distinct types of metabolic dysfunction-associated steatotic liver disease. Nat. Med..

[49] Giardoglou P., Gavra I., Amanatidou A.I., Kalafati I.P., Symianakis P., Kafyra M., Moulos P., Dedoussis G.V. (2024). Development of a Polygenic Risk Score for Metabolic Dysfunction-Associated Steatotic Liver Disease Prediction in UK Biobank. Genes.

