# Supplementary material for: The Protective Effect of FOXO3 rs2802292 G-Allele on Food Intake in a Southern Italian Cohort Affected by MASLD
Source: Nutrients. 2025 Apr 10;17(8):1315. doi: 10.3390/nu17081315 (PMC12030307; doi:10.3390/nu17081315)
Supplement: Supplementary file 1 [file nutrients-17-01315-s001.zip › nutrients-3516973-Supplementary Materials.pdf]

nutrients

MDPI

SUPPLEMENTARY MATERIALS

Table S1. Single food from the FFQ (EPIC) and relative the food grouping used for the analyses.

Copyright: © 2025 by the authors. Licensee MDPI, Basel, Switzerland. This article is an open access article distributed under the terms and conditions of the Creative Commons Attribution (CC BY) license (https://creativecommons.org/licenses/by/4.0/).

Food Group	Sample foods
1) Dairy	Grated cheese; Fontina; Pecorino/Caciotta cheese; Emmental/Groviera cheese; Caciocavallo cheese; Parmesan cheese; Other aged cheeses; Sliced melted cheese; Spreadable cheese; Crescenza cheese, Mozzarella cheese, Taleggio cheese; Gorgonzola cheese; Robiola cheese; Other soft cheeses; Whole milk coffee; Whole yougurt; Fruit yogurt; White whole milk; Cappuccino
2) Low Fat Dairy	Partially skimmed coffee-milk; Skimmed-Partly Skimmed Milk; Cottage Cheese; Low-fat yoghurt
3) Eggs	Eggs
4) White Meat	White stewed meat; White roast; White schnitzel; White sliced meat; White steak; White hamburger; White meatballs; Chicken leg; Chicken breast; Other chicken parts; Rabbit; Other types of white meat
5) Red Meat	Meat broth; Red stewed meat; Red roast; Boiled meat; Red schnitzel; Red sliced meat; Red steak; Red hamburger; Red meatballs; Pork; Sausages; Liver; Offal; Other red meats
6) Fish	Hake; Sole; Sardines; Trout; Swordfish; Other types of fish
7) Seafood/Shellfish	Praws; Octopus/Cuttlefish; Mussels/Clams
8) Leafy Vegetables	Salad; Spinach
9) Fruiting Vegetables	Tomatoes in season; Tomatoes out of season; Raw peppers; Peppers; Eggplants
10) Root Vegetables	Raw carrots; Cooked carrots; Beets
11) Other Vegetables	Minestrone; Raw artichokes/celery; Cooked artichokes; Cooked mushrooms; Cooked onions; Broccoli; Brussels Sprouts; Black cabbage; Cauliflower; Turnip greens; Savoy cabbage
12) Herbes & Spices	Onions
13) Legumes	Legume soap; Beans; Peas
14) Potatoes	Potatoes
15) Fruits	Apple; Pear; Banana; Kiwi; Orange/Grapefuit; Tangeerine; Grapes; Peach; Apricot; Plum; Strawberry; melon; Fruit salad; Grapefruit
16) Nuts	Walnuts/Nuts
17) Grains	Pasta; Pasta with tomato; ragù; Pasta with other sauces; White rice; Riso/Risotti; Other risottos; Soup pasta; Polenta; White bread; Whole wheat bread, Other typer of bread

18) Olive Oil	Olive oil; Olive oil in the sauce; Olive oil in the meat sauce; Olive oil in minestrone; Olive oil on vegetables; Olive oil on boiled vegetables; Olive oil on vegetables on white meat; fried olive oil
19) Seed Oil	Peanut oil on raw vegetables; Sunflower oil on row vegetables; Corn oil on raw vegetables; Soybean oil on raw vegetables; Other seed oils on raw vgetables; Seed oil on white meat; Seed oil in sauce; Seed oil in meat sauce; Seed oil in minestrone; Peanut oil on boiled vegetables; Sunflower oil on boiled vegetables; Corn oil on boiled vegetables; Soybean oil on boiled vegetables; Other seed oils on boiled vegetables; Seed oil on stewed meat; Peanut oil on stewed meat; Sunflower oil on stewed meat; Corn oil on stewed meat; Soybean oil on stewed meat; Other seed oils on stew meat; Non-fried seed oil; Fried peanut oil; Fried sunflower oil; Fried corn oil; Fries corn oil; Fried soybean oil; Other fries seed oil
20) Other Dressing Fats	Stock cube; Butter; Margarine; Butter in the sauce; Margarine in the sauce; Butter in the meat sauce; Margarine ragout; Butter in minestrone; Additional butter; Animal fat; Mayonnaise; Butter; Butter in stew meat; Margarine in stew meat; Fried butter; Fried margarine
21) Sweets	Rusks; Cookies; Brioches; Jam; Hazelnut cream; Stuffed cake; Unstuffed cake; Spoon cake; Dry pastries; Stuffed pastries; Chocolate; Candy; Summer ice cream; Winter ice cream
22) Added Sugars	Sugar in milk; Sugar in coffee; Sugar in yogurt; Sugar in milk with coffee; Sugar in cappuccino; Sugar in tea; Sweetener; Honey
23) Juices	Orange juice; Fruit juices
24) Caloric Drink	Soft drink
25) Ready-to-Eat Dish	Slice of pizza; Pizza from pizzeria, Pizza at home; Flas; Sandwich with cold cuts; Cheese sandwich; Sandwiches with vegetables; Appetizer in oli; Appetizer in vinegar
26) Coffee & The	Decaffeinated coffee; Bar espresso coffee; Mocha coffee; Other types of coffee; Tea
27) Wine	Red wine; White wine
28) Beer	Beer
29) Spirits	Wine/liqueur aperitifs; Spirits
Processed Food	
30) Eggs	Omelette
31) Meat	Canned meat; Salami; Mortadella; Bresaola; Soppressata; Other types of cured meats; Ham fat; Cooked ham; Raw ham
32) Fish	Preserved fish; Canned fish
33) Grains	Egg pasta; Stuffed pasta; Sandwiches; Breadsticks

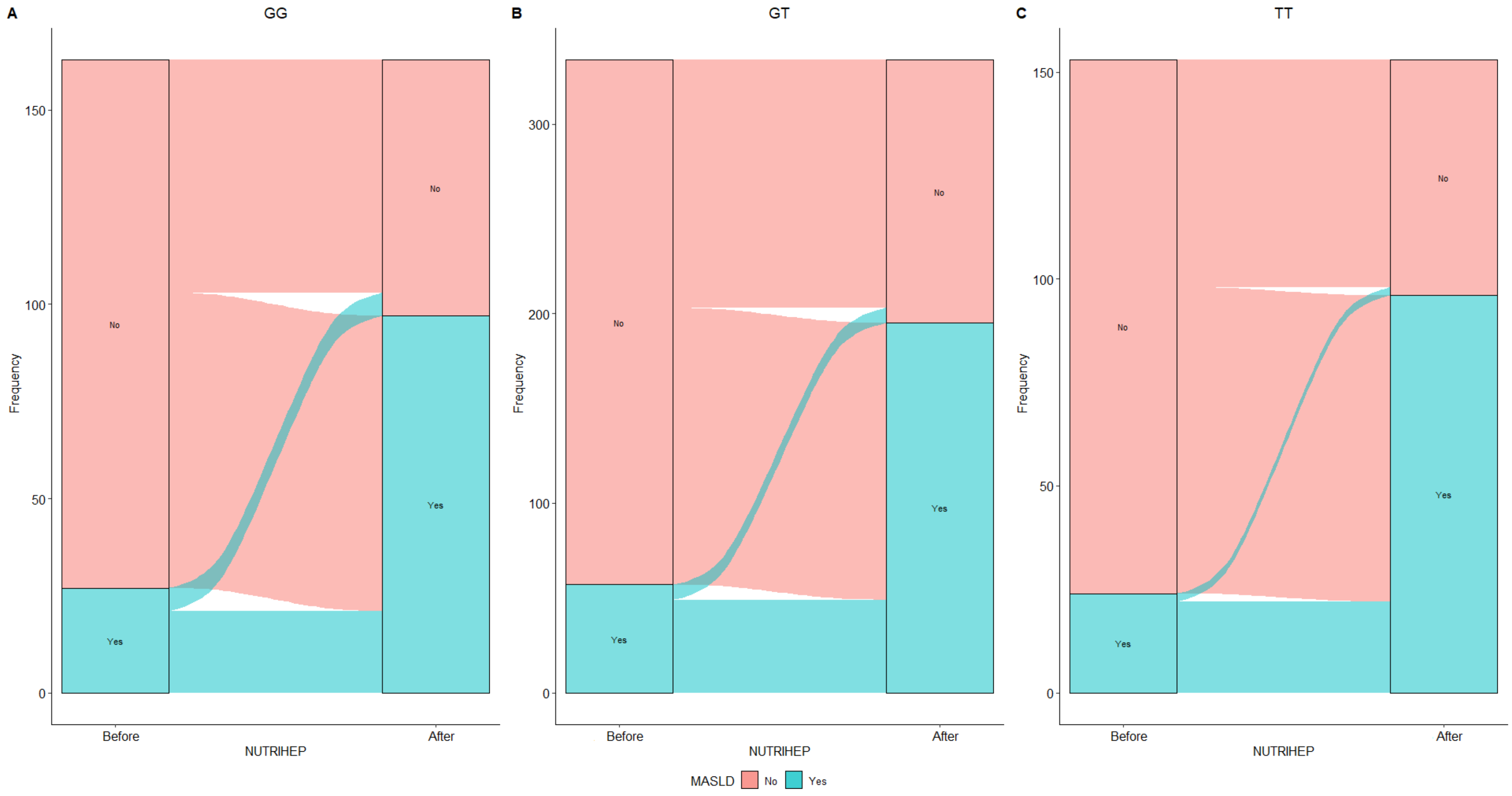

Figure S1. Alluvial plot of MASLD condition in the genotypes categories.

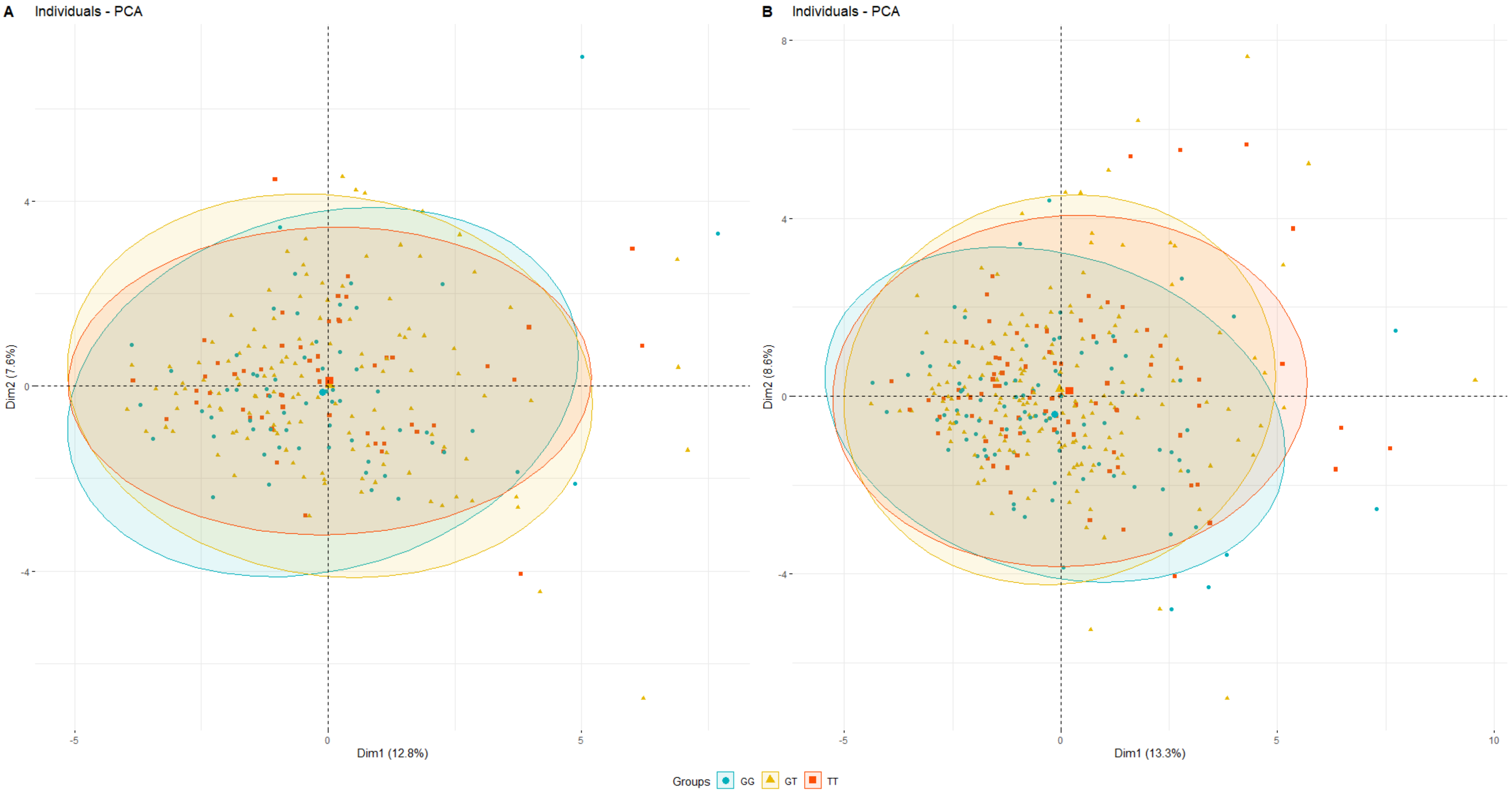

Figure S2. Biplot of PCA on the food group intake showing the projection of the data set on the PC1 × PC2 plane, stratified for genotypes (GG, GT, and TT) in subjects without MASLD (A), and with MASLD (B).

Disclaimer/Publisher's Note: The statements, opinions and data contained in all publications are solely those of the individual author(s) and contributor(s) and not of MDPI and/or the editor(s). MDPI and/or the editor(s) disclaim responsibility for any injury to people or property resulting from any ideas, methods, instructions or products referred to in the content.
